# Global CpG DNA Methylation Footprint in Kaposi’s Sarcoma

**DOI:** 10.3389/fcimb.2021.666143

**Published:** 2021-07-09

**Authors:** Guy Journo, Anuj Ahuja, David Dias-Polak, Yonatan Eran, Reuven Bergman, Meir Shamay

**Affiliations:** ^1^ Daniella Lee Casper Laboratory in Viral Oncology, Azrieli Faculty of Medicine, Bar-Ilan University, Safed, Israel; ^2^ Department of Dermatology, Rambam Health Care Campus, Haifa, Israel

**Keywords:** Kaposi’s sarcoma, CpG methylation, KS, KSHV, gene expression, promoter, enhancer

## Abstract

Kaposi’s sarcoma-associated herpesvirus (KSHV), also familiar as human herpesvirus 8 (HHV-8), is one of the well-known human cancer-causing viruses. KSHV was originally discovered by its association with Kaposi’s sarcoma (KS), a common AIDS-related neoplasia. Additionally, KSHV is associated with two B-lymphocyte disorders; primary effusion lymphoma (PEL) and Multicentric Castlemans Disease (MCD). DNA methylation is an epigenetic modification that is essential for a properly functioning human genome through its roles in chromatin structure maintenance, chromosome stability and transcription regulation. Genomic studies show that expressed promoters tend to be un-methylated whereas methylated promoters tend to be inactive. We have previously revealed the global methylation footprint in PEL cells and found that many cellular gene promoters become differentially methylated and hence differentially expressed in KSHV chronically infected PEL cell lines. Here we present the cellular CpG DNA methylation footprint in KS, the most common malignancy associated with KSHV. We performed MethylationEPIC BeadChip to compare the global methylation status in normal skin compared to KS biopsies, and revealed dramatic global methylation alterations occurring in KS. Many of these changes were attributed to hyper-methylation of promoters and enhancers that regulate genes associated with abnormal skin morphology, a well-known hallmark of KS development. We observed six-fold increase in hypo-methylated CpGs between early stage of KS (plaque) and the more progressed stage (nodule). These observations suggest that hyper-methylation takes place early in KS while hypo-methylation is a later process that is more significant in nodule. Our findings add another layer to the understanding of the relationship between epigenetic changes caused by KSHV infection and tumorigenesis.

## Introduction

Kaposi’s sarcoma-associated herpesvirus (KSHV), also familiar as human herpesvirus 8 (HHV- 8), belongs to the γ-herpesvirus family and is one of the well-known human cancer-causing viruses (Chang et al., 1994; Purushothaman et al., 2016). KSHV was originally discovered by its association with Kaposi’s sarcoma (KS) (Chang et al., 1994), a common AIDS-related neoplasia of endothelial/mesenchymal origin (Boshoff et al., 1995; Li et al., 2018). Additionally, KSHV is associated with two B-lymphocyte disorders; primary effusion lymphoma (PEL) and Multicentric Castlemans Disease (MCD), which are characterized by proliferation of B-cells in body cavities and lymph nodes, respectively (Parravicini et al., 2000; Henke-Gendo and Schulz, 2004). Similar to other herpes viruses, KSHV infection can be characterized as lytic or latent. During lytic replication, virions are assembled and released from the cell. This process requires DNA synthesis together with expression of virion structural protein genes and results in death of the infected cell. Latent infection, however, is characterized by the persistence of the viral genome as a covalently closed circular episome DNA with very limited viral gene expression. In KS most of the cells are latently infected, but the few cells that turn on the lytic phase express lytic proteins with critical roles in the pathogenesis (Ganem, 2006; Zhang et al., 2015). KS is a low-grade vascular tumor that can involve the skin, mucosa, and viscera. There are four different epidemiologic-clinical forms of KS; Classic, Endemic, Immunosuppression-Associated and AIDS-Associated KS (Antman and Chang, 2000). In recent years there have been several advancements in our understanding of KS including promising targeted therapeutic agents, but despite these advances, KS still remains the most prevalent malignancy among patients with AIDS and continues to plague patients with drug-related or transplant-associated immunosuppression.

DNA methylation is an epigenetic modification that is essential for a properly functioning human genome through its roles in chromatin structure maintenance, chromosome stability and transcription regulation. Expressed promoters tend to be un-methylated whereas methylated promoters tend to be inactive (Antequera et al., 1990; Baylin, 2005). DNA methylation involves the transfer of a methyl group to cytosine in a CpG dinucleotide by DNA methyltransferases which create or maintain methylation patterns (Bestor, 2000). In mammalian cells, DNA methylation is added and maintained by a few DNA methyltransferases (DNMTs); DNMT1, DNMT3A and DNMT3B. DNMT1 acts as the “maintenance” methyltransferase, due to its preference for hemi-methylated DNA, which is abundant following DNA replication. DNMT3A and DNMT3B, often referred to as “*de-novo*” methyltransferases, are responsible for establishing patterns of DNA methylation. While enzymes that catalyze DNA methylation have been thoroughly studied, the enzymes and mechanisms of DNA de-methylation have remained elusive until recently. The TET (ten-eleven translocation) family proteins, has the ability to catalyze sequential oxidation reactions; converting 5-mC first to 5-hydroxymethylcytosine (5-hmC), then 5-formylcytosine, and finally 5-carboxylcytosine (5-caC) (Huang et al., 2014; Putiri et al., 2014). A following decarboxylation of 5-caC, by either a thymine-DNA glycosylase or other DNA repair enzyme, leads to removal of the methylated nucleotide and results in DNA de-methylation (Ito et al., 2010).

Few studies have shown the ability of KSHV to alter the methylation levels of specific cellular gene promoters. Transcription repression *via* CpG DNA hyper-methylation of p16INK4a (*CDKN2A*) (Platt et al., 2002), the TGF-beta type II receptor (*TbetaRII, TGFBR2*) (Di Bartolo et al., 2008), and PDZ-LIM domain-containing protein 2 (*PDLIM2*) (Sun et al., 2015) promoters have been detected in KSHV-infected primary effusion lymphoma (PEL) lines. Hyper-methylation of the H-cadherin (*CDH13*) promoter has been reported in LANA expressing endothelial cells and PEL (Shamay et al., 2006). In a previous study (Journo et al., 2018), we revealed the global CpG DNA methylation in KSHV-positive PEL cells and *de-novo* infected B cell line (BJAB219). Following KSHV infection, the virus imposes global hyper-methylation on the cellular genome while extensive global hypo-methylation seems to occur later on as cancer progresses to PEL. In a recent study (Naipauer et al., 2020), we have found global hypo-methylation and up regulation of tumor-driving genes during the process of KSHV dependent transformation in a mouse model for KSHV sarcomagenesis.

These previous studies in *de-novo* infected B-cells, KSHV-sarcomaregnesis mice model, and PEL suggested a wave of hyper-methylation following KSHV-infection, and hypo-methylation during the development of KSHV-associated transformation. In this study we were interested to reveal the methylome of the most frequent KSHV-associated malignancy, KS. To answer this question, we performed a global methylation analysis in KS and normal skin biopsies. Interestingly, we found that the percentage of hyper-methylation in KS is very similar to PEL, while hypo-methylation is very different. Furthermore, methylation and specifically hypo-methylation can differentiate plaque from nodule.

## Materials and Methods

### Patient Samples

The Kaposi’s sarcoma tissues and normal controls were reviewed and ethically approved by the institutional Helsinki committee at Rambam hospital (number 0391-15-RMB). Written informed consent was obtained by all participants. Punch biopsies of 4 mm were taken, and DNA was isolated using DNeasy Blood & Tissue Kit (QIAGEN) according to manufacturer procedure.

### DNA Isolation and Illumina MethylationEPIC BeadChip

Genomic DNA was isolated from cells using DNeasy Blood & Tissue Kit (QIAGEN). Next, gDNA samples were bisulfite converted (D5001 Zemo), and then hybridized to MethylationEPIC BeadChip (Illumina) according to manufacturer’s protocol. The BeadChip array was performed in a single-base extension reaction, stained and imaged on an Illumina HiScan. The raw data was exported from GenomeStudio and normalized using ChAMP R pipeline (Morris et al., 2014) that has the ability to run a series of programs in which the output of one program is used as an input to the next one. The different programs decrease biases from known technical issues, such as adjustment for type I and type II probes, background correction and batch effects between chips. Statistical analysis was performed using limma program within ChAMP pipeline and JMP-genomic software. The methylation analysis was performed at the Genomics Core Facility, BioRap Technologies (Rappaport Research Institute, Technion).

### DNA Methylation Data Analysis

Methylation rates of selected CpG sites were calculated (using GenomeStudio Methylation Module Software) as methylation β-value ranging from 0 (completely un-methylated) to 1 (completely methylated). Probes with a detection P-value of over 0.05 or blank β-value were excluded from further analyses. Differences in β-values (Δβ) between KS and control samples were determined as Δβ≥0.25 and Δβ≤-0.25 and named as hyper and hypo-methylated, respectively (Kulis et al., 2015). To determine if these differentially methylated CpG are located in regulatory regions, genomic regions 100 bp up-stream and downstream of each CpG were analyzed in EnhancerAtlas (http://www.enhanceratlas.org/index.php) (Gao and Qian, 2020). To reduce the effect of redundant probes in the same regulatory region, probes residing within 200 bp were merged into a single interval using MergeBED (Galaxy bedtool, https://usegalaxy.org/) before the analysis on GREAT (http://great.stanford.edu/public/html/index.php) (McLean et al., 2010) to identify common functional processes and phenotypes. To generate the final lists of differentially methylated enhancers, two or more CpGs that were differentially methylated in the same enhancer region (up to 1000 bp apart) were merged using MergeBED. In the case of gene promoters, the final lists were based on the identified gene names. Gene-ontology analysis was performed on DAVID Bioinformatics Resources 6.8 (https://david.ncifcrf.gov/home.jsp) (Huang da et al., 2009).

### Data Access

Global methylation analysis obtained from the MethylationEPIC BeadChip (Illumina) analyses are available at: http://biodb.md.biu.ac.il/biu/shamay_lab_data.html.

## Results

### Mapping the Human Methylome in Kaposi’s Sarcoma

**Figure 1:**
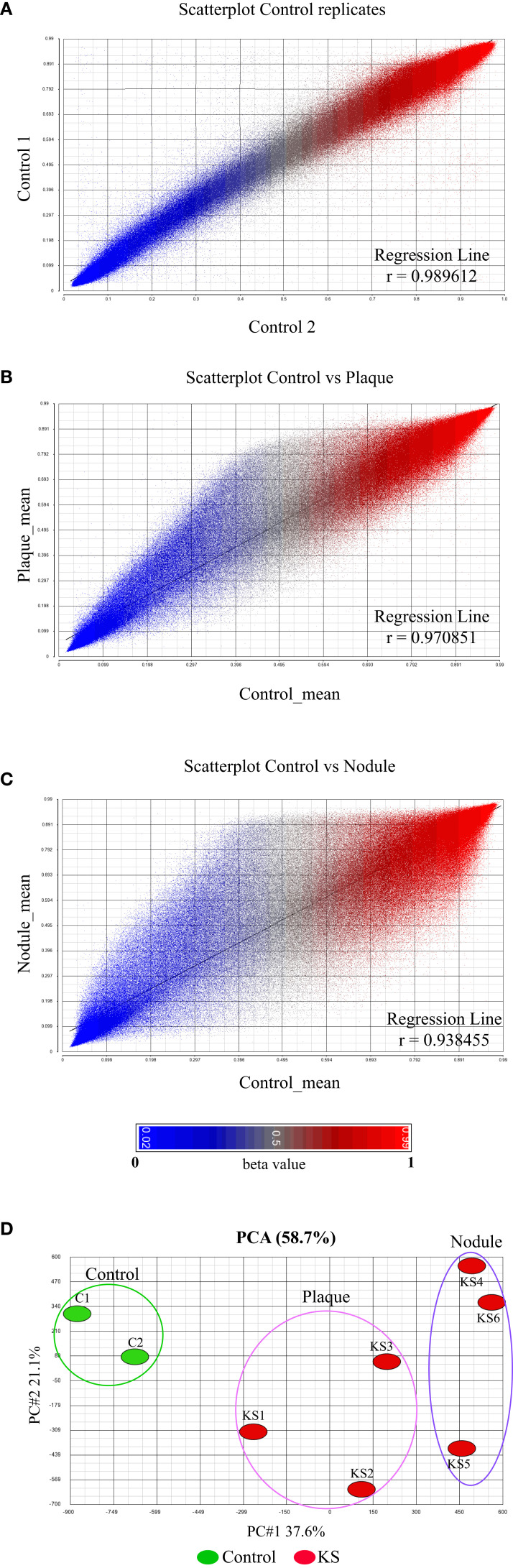
Global DNA methylation clearly distinguishes normal skin from KS. **(A–C)** The raw β value (presented between 0 and 1) of each CpG probe from the 850K BeadChip is represented by a single dot. Correlation coefficient analysis: **(A)** between biological replicates of uninfected skin controls showing close identity between replicates and **(B, C)** between uninfected skin control and KSHV infected KS samples at early tumor stage [Plaque **(B)**] and late tumor stage [Nodule **(C)**]. The raw β value (presented between 0 and 1) of each CpG probe from the 850K BeadChip is represented by a single dot. **(D)** Principal component analysis of the raw β values from the 850K BeadChip showing the variability between control and KS samples.

We have previously revealed the global methylation footprint in PEL cells and found that many cellular gene promoters become differentially methylated and hence differentially expressed in KSHV chronically infected PEL cell lines (Journo et al., 2018). PEL is originated from B cells and therefore might present a distinct methylation footprint from KS that is originated from either endothelial or mesenchymal cells (Boshoff et al., 1995; Li et al., 2018). We were interested to reveal the methylome of the most frequent KSHV-associated malignancy. For this analysis, we obtained six classic KS skin biopsies; three plaques (less progressed disease) and three nodules (more progressed disease), and two additional control skin biopsies. DNA isolated from skin biopsy was sheared, bisulfite treated and hybridized to the MethylationEPIC BeadChip. The EPIC array has the ability to cover over 850,000 CpG sites throughout the human genome, and every CpG site analyzed receives a methylation score known as beta value between 0 (un-methylated) and 1 (fully methylated) (Pidsley et al., 2016). The results of regression analysis of the raw beta values between the control samples detected correlation coefficient of r>0.989 (**Figure 1A**). The tight correlation between our two controls gave us confidence of the quality and reproducibility of our sample collection and methylation analysis. Regression analysis of the controls and plaques clearly shows methylation differences with hyper-methylation in plaque more pronounced (**Figure 1B**). Regression analysis of the controls and nodules shows more profound methylation differences with more extensive hyper-methylation and significant number of hypo-methylated probes (**Figure 1C**). We then applied all eight samples on a principal component analysis (PCA) of the global methylation beta values (**Table S1**). This analysis clearly differentiates KS from normal skin (**Figure 1D**). It seems that the methylation pattern reflects the progression of KS from plaque to nodule, since the nodule is distributed further away from the control, than the plaque.

### KS Disease Progression Can Be Determined by Global Hypo-Methylation Footprint

**Figure 2:**
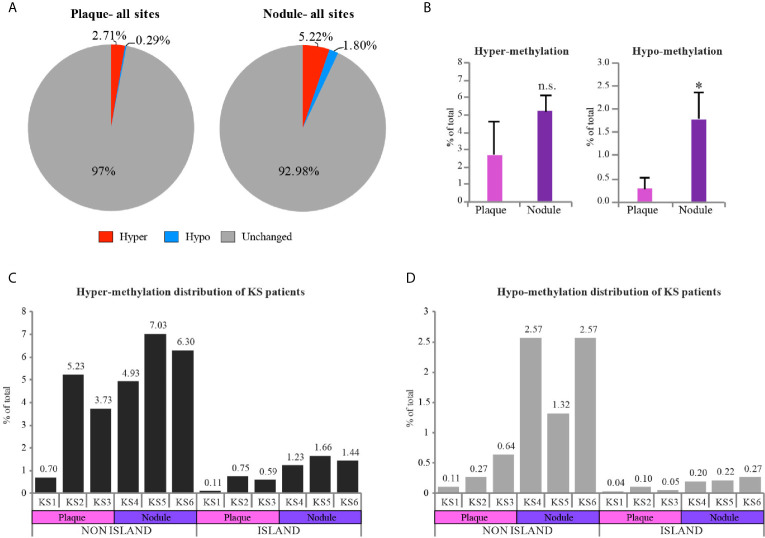
Hypo-methylation increases as KS progresses from plaque to nodule. **(A)** Pie charts represent the percentage of hyper and hypo-methylated CpGs relative to control skin in two different stages of disease (Nodule and Plaque). **(B)** Column charts represent hyper and hypo-methylation distribution of Plaque vs. Nodule compared to control (represented as mean of three samples +SD). **(C, D)** Column charts represent hyper and hypo-methylation distribution of KS samples compared to control in CpG island vs. non CpG island regions. Percentage of each individual sample is indicated above each column. Hyper and hypo-methylation threshold was set as Δβ≥0.25 and Δβ≤-0.25 respectively. Two-tailed T-tests were performed. *p < 0.05; n.s., not significant.

Looking deeper into the PCA (**Figure 1D**), we noticed one sample, plaque KS1, that represents an intermediate state between KS and normal skin, while the rest of the KS samples are distributed further away from the control skin. This might be explained due to the early stage (plaque) of sample KS1, a stage where infected cells are not always the major population within the lesion. We took this into consideration while further analyzing our data. We next decided to create a pie chart showing the percentage of hyper and hypo-methylated CpGs relative to control in two different stages of disease (Nodule and Plaque) (**Figure 2A**). This analysis revealed 2.71% (3.77% when sample KS1 was omitted) hyper-methylated and 0.29% (0.38% when sample KS1 was omitted) hypo-methylated sites in plaque, and 5.22% hyper-methylated and 1.8% hypo-methylated sites in nodule. While the hyper-methylation increased from 2.71% to 5.22% (1.9 fold), hypo-methylation increased from 0.29% to 1.8% (6.2 fold) during the process from plaque to nodule (**Figure 2B**). This observation suggests that hyper-methylation takes place early in KS while hypo-methylation is a later process that is more significant in nodule. When the probes were divided to those located within CpG-islands and non-island we have found significant preference for non-island over island in both hyper and hypo-methylated sites (**Figures 2C, D**). Interestingly, focusing solely on hypo-methylation of the non-CpG island sites, revealed a clear-cut difference between plaque and nodule (**Figure 2D**). Altogether, this supports the notion that hyper-methylation takes place in early stages of KS and accumulates as disease progresses, while hypo-methylation is a better indicator of disease progression, and this phenomenon is more substantial in non-CpG sites.

### Differentially Methylated Regulatory Elements in KS

**Figure 3:**
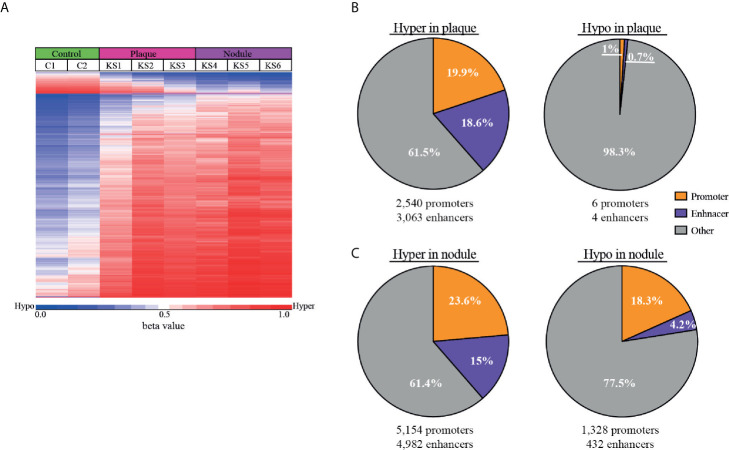
Hyper-methylation in KS is prevalent in enhancer regions. **(A)** Heat map of the most differentially methylated CpG sites (raw β values) comparing control and KS samples. Hyper (red) and hypo (blue) methylation threshold was set as Δβ≥0.25 and Δβ≤-0.25 respectively with FDR ≤ 0.05. **(B, C)** Pie charts representing the percentage of hyper and hypo-methylated CpGs that are located within cellular promoters (orange) and enhancers (purple) for plaque **(B)** and nodule **(C)**. The number of cellular enhancers and promoters identified in our analysis are presented below.

The most differentially methylated probes between normal skin and KS are presented in a heatmap (**Figure 3A**), where the most changes are towards hyper-methylation in KS. At the top of the heatmap a small number of probes turn hypo-methylated, a phenomenon more profound in the nodule samples. To obtain deeper understanding of the methylation changes in KS, we identified differentially methylated CpGs in regulatory elements. Therefore, differentially methylated CpGs (a genomic region 100 bp up-stream and downstream of each CpG) were analyzed in EnhancerAtlas (http://www.enhanceratlas.org/index.php) (Gao and Qian, 2020). Out of the 19,242 hyper-methylated CpG in plaque, 3,838 (19.9%) CpG were located in 2,540 promoters and 3,577 (18.6%) were located in 3,063 enhancers. Out of the 580 hypo-methylated CpG in plaque, 6 (1%) were located in 6 promoters and 4 (0.7%) were in 4 enhancers. Analysis in nodule revealed that out of the 40,158 hyper methylated CpG, 9,477 (23.6%) were located in 5,154 promoters and 6,041 (15%) were located in 4,982 enhancers. Out of the 11,446 hypo-methylated CpG in nodule, 2,092 (18.3%) were located in 1,328 promoters and 485 (4.2%) were located in 432 enhancers (**Figures 3B, C** and **Table S2, S3**). In many cases more than one methylated probe was located in the same promoter/enhancer, this is the reason that the number of differentially methylated promoters/enhancers are smaller than the total number of probes within these elements. Several of the hyper-methylated promoters we identified in KS are among the few promoters that were previously reported and confirmed as hyper-methylated in KSHV-infected cells, such as the transforming growth factor–β type II receptor (TGFBR2/TβRII) (Di Bartolo et al., 2008), and EH Domain Containing 3 (EHD3) (Journo et al., 2018) in PEL, and Cadherin 13 (CDH13) (Shamay et al., 2006) in LANA expressing endothelial TIME cells. While around 20% of both hypo and hyper methylated CpGs in nodule are located in promoters, the enhancers are preferentially hyper-methylated (15%) relative to only 4% in hypo-methylation. This also revealed that more hypo-methylation changes are outside regulatory elements, and towards intergenic regions and gene body. This phenomenon is even more profound in plaque, where less than 2% of the hypo-methylation takes place in promoters and enhancers.

The 5,154 promoters and 4,982 enhancers which were hyper-methylated, and 1,328 promoters and 432 enhancers which were hypo-methylated in nodule were analyzed on GREAT (http://great.stanford.edu/public/html/index.php) (McLean et al., 2010) to identify common functional processes (**Table S4**) and phenotypes (**Table S5**). Biological processes in hyper-methylated promoters include cornification, establishment of skin barrier, hemiendosome assembly, and cell surface junction. Biological processes in hyper-methylated enhancers include regulation of mitochondrial membrane permeability, apoptotic signaling, keratinocytes differentiation, skin development, hair follicle development, and neuronal death. Hyper-methylated promoters include genes associated with phenotypes of abnormal keratinocyte differentiation, blistering and scaly skin. Hyper-methylated enhancers include genes associated with phenotypes of dermal atrophy, abnormal skin, hypotrichosis, nail dystrophy, epidermal acanthosis, abnormal epidermis stratum, thick epidermis, and abnormal wound healing. Altogether it seems that hyper-methylated genes control abnormal skin morphology, a characteristic phenotype of KS.

Biological processes in hypo-methylated promoters include regulation of immune response, leukocyte and lymphocyte activation, T-cell receptor, MHC I/II protein complex, and intermediate filaments. Biological processes in hypo-methylated enhancers include regulation of cell migration and motility, wound healing, angiogenesis, and epithelial to mesenchymal transition (EMT). Hypo-methylated promoters include genes associated with phenotypes of abnormality of lymphocytes, leukocytes, B-cell physiology, T-cell physiology, immunoglobulin levels, humoral immunity, neutrophil physiology, and lymph node physiology. Hypo-methylated enhancers include genes associated with phenotypes of abnormal blistering of the skin, angiogenesis, plasma cell number, lymph node morphology, second pharyngeal arch, small pharyngeal arch, and spine curvature. Altogether it seems that hypo-methylated genes control immune response phenotype, and genes that should be methylated in cells that are not pharyngeal or spine. Considering the important role of the EMT pathway (called EndMT in endothelial cells) in KS development, our study indicates that DNA methylation participate in the activation of this pathway by KSHV.

### Common CpG Methylation in PEL and KS

While the cell type of PEL is very different from KS, we were interested to see which of the methylation changes are common. Therefore, we compared the differentially methylated CpG between PEL (Journo et al., 2018) and KS and identified 199 hyper-methylated and 236 hypo-methylated promoters (**Tables S6**, **S7**). Gene-ontology analysis on DAVID Bioinformatics Resources 6.8 (https://david.ncifcrf.gov/home.jsp) (Huang da et al., 2009) for common hyper-methylated promoters identified alternative splicing, cell membrane, SH2 domain, tyrosine protein phosphatase, transcriptional repressor activity, transcriptional misregulation in cancer, and homeobox (**Table S8**). Several regulators of TGF-beta signaling were identified, such as TGFBR2, SMAD9, NEDD4L, and TGIF1. As mentioned above, TGFBR2 has been shown previously as hyper-methylated and repressed in KSHV-infected cells (Di Bartolo et al., 2008). In a previous study (Journo et al., 2018) we have found that several *DUSP* genes, such as *DUSP5*, *DUSP6*, and *DUSP10* were repressed and their promoter was hyper-methylated in PEL. Interestingly we found that *DUSP2, DUSP6, DUSP7, DUSP13, DUSP16*, and *DUSP28* were hyper-methylated in KS. Since DUSPs are dual specificity phosphatases that dephosphorylate MAPK, ERK, JNK and p38, their down-regulation in KSHV-infected cells may contribute to the activation of these kinases by KSHV. Gene-ontology analysis for common hypo-methylated gene promoters identified glycosylation, cell adhesion, plasma membrane, immunoglobulin, cellular defense response, and homeobox (**Table S8**). In addition, several GTP-binding superfamily and immuno-associated nucleotide (IAN) subfamily of nucleotide-binding proteins, GIMAP1, GIMAP4, GIMAP5, GIMAP7, and GIMAP8. The large number of proteins associated with cell adhesion, plasma membrane and immunoglobulin indicates that methylation changes within gene promoters have profound effect on the cell surface of KSHV-associated malignancies.

### CpG Methylation and Gene Expression in KS and PEL

**Figure 4:**
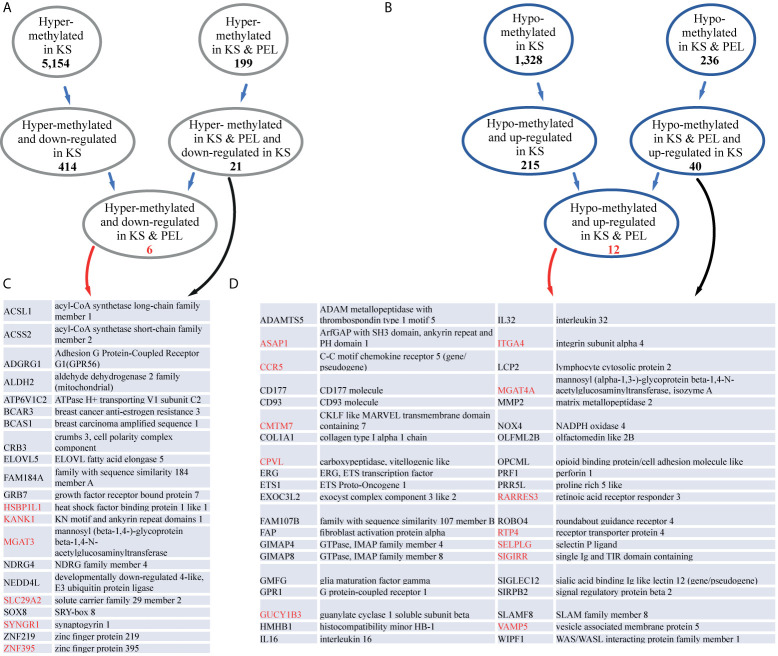
KS and PEL share common differentially methylated promoters. **(A)** The number of hyper-methylated promoters common in KS and PEL, and down-regulated are indicated. **(B)** The number of hypo-methylated promoters common in KS and PEL, and up-regulated are indicated. **(C)** Gene names and their descriptions for hyper-methylated promoters common in KS and PEL, and down-regulated in KS are indicated. Genes that are also down-regulated in PEL are indicated in red. **(D)** Gene names and their descriptions for hypo-methylated promoters common in KS and PEL, and up-regulated in KS are indicated. Genes that are also up-regulated in PEL are indicated in red.

Next, we correlated the methylation changes we observed with gene expression. We intersected the differentially methylated gene promoters with a previous RNA-sequencing (RNA-seq) analysis performed on KS (Tso et al., 2018). Within the list of 5,154 hyper-methylated promoters, 414 were down-regulated in KS (**Figure 4A** and **Table S9**). On the more restricted list of 199 hyper-methylated promoters both in KS and PEL, 21 were down-regulated in KS (**Figure 4C**). Of these 21 genes, five are involved in metabolic pathways (ACSL1, ACSS2, ALDH2, ATP6V1C2, MGAT3) seven in acetylation (ACSL1, ACSS2, ALDH2, ELOVL5, BCAR3, NEDD4L, SYNGR1) and three are transcription factors (ZNF219, ZNF395, SOX8). We identified six genes that were hyper-methylated and repressed in both KS and PEL (**Figure 4C**).

Among the 1,328 hypo-methylated promoters, 215 were up-regulated in KS (**Figure 4B** and **Table S9**). On the more restricted list of 236 hypo-methylated promoters both in KS and PEL, 40 were up-regulated in KS, and 12 were up-regulated both in KS and PEL (**Figure 4D**). Of the 40 up-regulated in KS 21 are glycoproteins (ADAMTS5, CCR5, CD177, CD93, GPR1, NOX4, SLAMF8, CPVL, COL1A1, FAP, ITGA4, MGAT4A, MMP2, OLFML2B, OPCML, PRF1, ROBO4, SELPLG, SIGLEC12, SIRPB2, SIGIRR), six of them contain immunoglobulin domain (SLAMF8, OPCML, ROBO4, SIGLEC12, SIRPB2, SIGIRR) and three are metallopeptidases (ADAMTS5, FAP, MMP2). Here again, many of the hypo-methylated and up-regulated genes encode for cell membrane proteins.

## Discussion

Here we present the cellular CpG DNA methylation in KS, the most common malignancy associated with KSHV. For this analysis we compared normal skin biopsy to KS samples of both plaque and nodule. The PCA analysis clearly differentiated normal skin from KS based on the cellular methylation data, indicating that indeed following KSHV infection and the development of KS there are significant changes in cellular DNA methylation. Moreover, these methylation changes can also differentiate less advanced disease (plaque) from more advanced disease (nodule). Comparison between normal skin and KS revealed that most changes are towards hyper-methylation. Hyper-methylation starts earlier, while hypo-methylation increases as KS progress from plaque towards nodule. Accumulation of hypo-methylation changes have been observed also in a previous study that followed tumor development of endothelial cells by KSHV (Naipauer et al., 2020).

Global cellular CpG methylation analysis in PEL detected ~ 6% hyper-methylation (5.99% in BCBL1 and 6.24% in BC3) (Journo et al., 2018). The percentage of hyper-methylation detected in KS is very similar, with 5.22% in nodule. The observation that already following *de-novo* infection KSHV induced 4.4% hyper-methylation (Journo et al., 2018), supports the notion that KSHV imposes hyper-methylation on the cellular genome shortly after infection. We found that many of the differentially methylated promoters in KS were different from PEL, this was expected taking into account that the methylation pattern in B-cells is different from endothelial/mesenchymal cells. Even though, we identified 199 promoters that become hyper-methylated in both KS and PEL, suggesting these promoters are hyper-methylated regardless of the type of the infected tissue.

As opposed to hyper-methylation, hypo-methylation is very different between PEL and KS, while in PEL we observed up to 30% hypo-methylation (27.5% in BCBL1 and 30.16% in BC3), only 1.8% was detected in KS. Analysis in Epstein-Barr virus (EBV) infected cells revealed a similar phenomenon; in infected B-cells the majority of changes were towards hypo-methylation (22.75% hyper- and 77.25% hypo-methylated) (Zhang et al., 2018), while in epithelial (gastric cancer EBV positive vs negative) cells the majority of changes were towards hyper-methylation (83.2% hyper- and 16.8% hypo-methylated) (Zhao et al., 2013). In the case of EBV infected B-cells DNMT1 and DNMT3B are downregulated, and DNMT3A is up-regulated (Leonard et al., 2011). While in EBV-positive epithelial cells high expression levels of DNMT1 and DNMT3B have been detected (Ksiaa et al., 2014). This change in DNMT expression along with changes in B-cell differentiation state might explain these changes. In KSHV infected PEL cells DNMT1 and DNMT3A were unchanged while DNMT3B was down-regulated (Journo et al., 2018). In KS vs normal skin the expression of DNMT1 and DNMT3B were unchanged while DNMT3A was up-regulated (Tso et al., 2018). The down-regulation of DNMTs in both EBV and KSHV infected B-cells might be one mechanism for the robust hypo-methylation observed in these cells.

The observation that 23.6% of the hyper-methylated CpGs were located in promoters and 15% were in enhancers, and 18.3% of the hypo-methylated CpGs were in promoters and 4.2% were in enhancers, suggests that these methylation changes might have an effect on cellular gene expression. Analysis of these promoters and enhancers on GREAT revealed that hyper methylated promoters and enhancers regulate genes associated with abnormal skin morphology a phenotype associated with KS development, while hypo-methylated promoters and enhancers regulate immune response genes and activation of B and T cells. We cannot exclude the possibility that some of these hypo-methylated promoter regions are the result of infiltrating immune cells into the lesion. Nevertheless, our study suggests that hypo-methylated enhancers regulate genes involved in EMT (in the case of endothelial cells called EndMT) an important process during the development of KS (Cheng et al., 2011; Gasperini et al., 2012), and might hint for the importance of methylation changes during KS development. To correlate methylation changes with gene expression, we intersected our methylation data with a published gene expression analysis in KS (Tso et al., 2018). We identified 414 hyper-methylated and repressed genes and 215 hypo-methylated and up-regulated genes in KS.

Most of the of the hyper- and hypo-methylated CpG are located at non-CpG island sites. This preference for non-CpG island sites was also observed in EBV infected cells (Zhang et al., 2018), but might results from the relatively small number of CpGs located in CpG-islands in the human genome and accordingly their relative representation in the MethylationEPIC BeadChip (Han et al., 2008; Pidsley et al., 2016). The differentially methylated promoters and enhancers identified here for KS, are only the first step in our understanding of CpG methylation in this tumor. Future studies should tackle the question why these specific promoters and enhancers become hyper-methylated in KSHV-infected cells.

## Data Availability Statement

The datasets presented in this study can be found in online repositories. The names of the repository/repositories and accession number(s) can be found below: http://biodb.md.biu.ac.il/biu/shamay_lab_data.html.

## Ethics Statement

The studies involving human participants were ethically approved by the institutional Helsinki committee at Rambam hospital (number 0391-15-RMB). The patients/participants provided their written informed consent to participate in this study.

## Author Contributions

Conceived and designed the experiments: GJ and MS. Performed the experiments: GJ, AA, and YE. Analyzed the data: GJ and MS. Contributed reagents/materials/analysis tools: DD-P and RB. Wrote the paper: GJ and MS. All authors contributed to the article and approved the submitted version.

## Conflict of Interest

The authors declare that the research was conducted in the absence of any commercial or financial relationships that could be construed as a potential conflict of interest.

## Supplementary Material

Supplementary Table 1 Differentially methylated CpGs.

Supplementary Table 2 Hyper methylated promoters and enhancers.

Supplementary Table 3 Hypo methylated promoters and enhancers.

Supplementary Table 4 Biological processes associated with differentially methylated regulatory elements.

Supplementary Table 5 Phenotypes associated with differentially methylated regulatory elements.

Supplementary Table 6 The common hyper-methylated gene promoters between KS and PEL.

Supplementary Table 7 The common hypo-methylated gene promoters between KS and PEL.

Supplementary Table 8 GO analysis.

Supplementary Table 9 KS methylation and expression.
