# Supplementary material for: Global CpG DNA Methylation Footprint in Kaposi’s Sarcoma
Source: Front Cell Infect Microbiol. 2021 Jul 9;11:666143. doi: 10.3389/fcimb.2021.666143 (PMC8300563; doi:10.3389/fcimb.2021.666143)
Supplement: Supplementary Table 6 — The common hyper-methylated gene promoters between KS and PEL. [file DataSheet_1.pdf]

Supplementary information:

Global CpG DNA methylation footprint in Kaposi's Sarcoma

Guy Journo, Anuj Ahuja, David Dias-Polak, Yonatan Eran, Reuven Bergman and Meir Shamay

Table S4: Biological processes associated with differentially methylated regulatory elements

HYPO ENHANCER	HYPO PROMOTER	HYPER ENHANCER	HYPER PROMOTER
**Biological process**	**Biological process**	**Biological process**	**Biological process**
regulation of cell migration	immune response	regulation of mitochondrial membrane	cornification
angiogenesis	regulation of immune system process	positive regulation of apoptotic signaling	hemidesmosome assembly
positive regulation of cell migration	regulation of immune response	centrosome localization	cell-substrate junction assembly
positive regulation of locomotion	defense response	positive regulation of protein localization to	establishment of skin barrier
positive regulation of cell motility	positive regulation of immune system process	response to insulin	regulation of water loss via skin
positive regulation of epithelial cell migration	leukocyte activation	positive regulation of protein insertion into mitochondrial membrane involved in apoptotic signaling pathway	
positive regulation of cellular component movement	regulation of leukocyte activation	regulation of keratinocyte differentiation cytoskeleton-dependent intracellular transport placenta development	
regulation of epithelial cell migration	regulation of cell activation	neuron death	
blood vessel morphogenesis	regulation of lymphocyte activation	regulation of stem cell population maintenance hair cycle	
positive regulation of endothelial cell	cell activation	negative regulation of cell-cell adhesion	
regulation of defense response	regulation of leukocyte cell-cell adhesion	hair cycle process	
negative regulation of intracellular signal transduction	regulation of T cell activation	organelle transport along microtubule cell junction assembly	
regulation of endothelial cell migration	positive regulation of leukocyte cell-cell	hair follicle development	
regulation of vascular endothelial growth factor receptor signaling pathway	positive regulation of cell activation lymphocyte activation	regulation of lipid biosynthetic process neuron apoptotic process	
vasculature development	positive regulation of leukocyte activation positive regulation of T cell activation positive regulation of cell-cell adhesion	skin epidermis development	
positive regulation of protein localization to	positive regulation of lymphocyte activation		
regulation of blood vessel endothelial cell	MHC protein complex		
positive regulation of epithelial to mesenchymal transition involved in	MHC class II protein complex		
response to laminar fluid shear stress	intermediate filament		
wound healing	integral component of lumenal side of endoplasmic reticulum membrane		
	tertiary granule		
	immunological synapse		
	intermediate filament cytoskeleton		
	alpha-beta T cell receptor complex		
	tertiary granule membrane		
	ficolin-1-rich granule membrane		
	cornified envelope		
	protein complex involved in cell adhesion		
	T cell receptor complex		
	extrinsic component of cytoplasmic side of plasma membrane		
	MHC class I protein complex		
**Molecular function**	**Molecular function**	**Molecular function**	**Molecular function**
phospholipid binding	antigen binding	cadherin binding	
growth factor binding	peptide antigen binding	repressing transcription factor binding	
platelet-derived growth factor receptor	CD4 receptor binding	cadherin binding involved in cell-cell adhesion	
interleukin-1 receptor activity	cytokine activity	protein binding involved in cell-cell adhesion	
	SH3/SH2 adaptor activity	histone demethylase activity (H3-K36	
	MHC protein binding		
	signaling adaptor activity		
	tumor necrosis factor receptor superfamily		

Table S5: phenotypes associated with differentially methylated regulatory elements

human phenotype	human phenotype	human phenotype	human phenotype
Abnormal blistering of the skin	Abnormality of lymphocytes	Nail dystrophy	Abnormal blistering of the skin
	Abnormality of leukocytes	Generalized abnormality of skin	
	Abnormality of cells of the lymphoid lineage	Dermal atrophy	
	Lymphopenia	Abnormality of the nail	
	Abnormality of B cell physiology	Anonychia	
	Recurrent bacterial infections	Reduced number of teeth Abnormality of epidermal morphology Abnormal number of	
	Leukocytosis	Ectodermal dysplasia	
	Abnormality of bone marrow cell morphology Abnormal immunoglobulin level	Nail dysplasia	
	Abnormality of humoral immunity	Epidermal acanthosis	
	Abnormal leukocyte count	Abnormality of the plantar skin of foot	
	Decrease in T cell count	Milia	
	Lymphoma	Abnormality of dental enamel Abnormality of odontoid tissue Laryngomalacia	
	Abnormality of the lymph nodes Inflammatory abnormality of the skin	Ectropion	
	Immunodeficiency	Hypotrichosis	
**mouse phenotype**	**mouse phenotype**	**mouse phenotype**	**mouse phenotype**
hemorrhage	abnormal cell-mediated immunity	abnormal epidermal layer morphology abnormal skin physiology	absent epidermis stratum corneum scaly skin
abnormal T cell physiology	abnormal adaptive immunity	abnormal epidermis stratum corneum	blistering
small second pharyngeal arch	abnormal immune cell physiology	abnormal epidermis stratum granulosum	abnormal keratinocyte differentiation
abnormal dendritic cell number	abnormal lymphocyte physiology	abnormal keratinocyte physiology	
small pharyngeal arch	abnormal T cell physiology	abnormal epidermis stratum spinosum	
absent pharyngeal arch arteries	abnormal immune serum protein physiology abnormal cytokine secretion	abnormal epidermis stratum basale morphology	
decreased myeloid dendritic cell number abnormal mononuclear phagocyte morphology decreased Langerhans cell	abnormal T cell activation	abnormal vibrissa morphology	
abnormal plasma cell number	abnormal lymphopoiesis	acanthosis	
absent second pharyngeal arch	abnormal leukopoiesis	abnormal keratinocyte morphology	
abnormal angiogenesis	abnormal B cell physiology	epidermis stratum spinosum hyperplasia	
abnormal myeloid dendritic cell morphology altered susceptibility to autoimmune disorder abnormal plasma cell morphology	abnormal T cell proliferation	shiny skin	
abnormal cytokine secretion	abnormal lymph node morphology	abnormal keratinocyte proliferation	
abnormal lymph node morphology	abnormal CD4-positive, alpha beta T cell	abnormal wound healing	
abnormal dendritic cell morphology	abnormal granulocyte physiology	abnormal epidermal-dermal junction	
abnormal spine curvature	decreased CD4-positive, alpha beta T cell number	increased integument system tumor incidence spontaneous skin ulceration dermal-epidermal separation	
abnormal cytokine level	abnormal CD4-positive, alpha beta T cell	thick epidermis	
	abnormal neutrophil physiology abnormal immunoglobulin level abnormal response to	abnormal tumor necrosis factor level	

Table S6: The common hyper-methylated gene promoters between KS and PEL

Hyper methylated in KS and PEL						
ABHD8	CHRNB1	FOXP2	KTN1	PHC2	SLC12A6	TGFBR2
ABR	CLIC5	FSD1	LOC728392	PLEKHA1	SLC16A3	TGIF1
ACBD7	CLIP4	FURIN	LPAR2	PLEKHF2	SLC1A1	TOX
ACE	CLSTN1	GALNT6	LPAR5	PLSCR2	SLC23A1	TRPV3
ACSL1	CMPK2	GFOD1	LPP	POU2F3	SLC29A2	TSPAN14
ACSS2	CPXM1	GNG13	LRRC14B	POU3F1	SLC2A5	UBE3A
ADAM19	CRB3	GPNMB	LRRC4	PRKAG2	SLC43A3	UNC13D
ADAP2	CRYL1	GPR114	LRRC56	PSAT1	SLC45A3	VASN
ADORA2A	CRYM	GPR160	LYPD6B	PSORS1C1	SLC6A12	WDR66
AGPAT1	CSNK1E	GPR56	LYSMD2	PTAFR	SLC6A6	WIZ
ALDH2	DBN1	GRB7	MAP3K14	PTP4A1	SLC7A7	ZBTB22
AMOTL1	DDAH1	GRN	MAP3K8	PTPN6	SLFNL1	ZCCHC11
AMPD3	DNAJC15	GTDC1	MAST4	PYCARD	SMAD9	ZNF219
APBB2	DPM2	HES5	MGAT3	RAB30	SMPDL3A	ZNF236
ARAP1	DUSP2	HOXA1	MICAL3	RAPGEF5	SNX13	ZNF395
ARPC1B	DUSP6	HOXA10	MMRN2	RARA	SNX25	ZNF563
ATP6V1C2	ECHDC1	HOXB8	MYL5	RASSF2	SORL1	ZNF582
B3GNT7	EHD3	HPS4	MYO18A	REPIN1	SOX8	ZNF664
BCAR3	ELOVL5	HSBP1L1	N4BP3	RFFL	SPOPL	ZNF860
BCAS1	EPB41	IFNGR2	NCF4	RGS7BP	SSH3	
BGLAP	ESPNL	ILDR1	NDRG4	RIMKLB	ST6GAL1	
BMF	ESRP1	ITGB7	NEDD4L	RIN1	STRA6	
C1orf100	ETS2	ITPRIP	NEK7	RNF13	SYNE2	
CALHM3	FAM134B	JDP2	NFKBIZ	RNF180	SYNGR1	
CAPG	FAM159A	KANK1	NLRC3	RNF43	SYTL2	
CBX5	FAM184A	KATNB1	NLRP2	RUNX3	TAL2	
CCDC102A	FAM26D	KCNQ1	OSBPL10	SEC14L1	TAS1R3	
CCDC62	FAM65B	KIAA0040	OSBPL6	SH3BGRL3	TCAP	
CCDC88B	FCGRT	KIAA0247	PBX4	SLA	TECTA	
CD81	FES	KLHDC7B	PDGFD	SLA2	TFF1	

Table S7: The common hypo-methylated gene promoters between KS and PEL

Hypo methylated in KS and PEL							
ADAMTS5	CD36	ERG	GREM2	LAMA2	NTM	ROBO4	TACC2
ADRA1A	CD6	ESR1	GRIK2	LCE5A	NUAK1	RPL13AP3	TBX3
AKAP12	CD93	ETS1	GTF2A1L	LCP2	OLFML2B	RTP4	TC2N
ALOX15	CDH17	EVX1	GUCY1B3	LMX1A	OPCML	SACS	TCF7L2
AMICA1	CDH3	EXOC3L2	HMHB1	LOC100190940	OPRM1	SCG3	TCN1
ANO1	CEBPE	EXT1	HOXC11	LRRC7	OR2B2	SCIN	TGM6
ANO3	CFLAR	FAM107B	HOXC13	LSP1	OR8B12	SCOC	TMEM200C
AQP1	CHN2	FAM170B	HOXC8	LTB4R	PCK1	SCPEP1	TMEM71
ASAP1	CHRNA3	FAP	HPCAL1	LZTFL1	PDCD1	SELPLG	TNFRSF10D
ATP10B	CLCA1	FBXO44	HSD17B2	MACC1	PDE4B	SEMA6D	TNN
AZU1	CLDN9	FCRL6	ICAM5	MAP2	PDYN	SERPINA4	TOX3
B3GALT1	CMTM7	FLJ12825	IL16	MGAT1	PIPOX	SERPINA5	TP63
BLVRA	COL12A1	FNDC3B	IL18RAP	MGAT4A	PLAC8	SERPINB6	TRAF3IP3
BMPR1B	COL16A1	FOXJ1	IL2RB	MLC1	PLD1	SH2D1B	TRIM40
BRF1	COL1A1	GABBR1	IL32	MLPH	POU6F2	SH3BP2	TTLL10
BTNL2	COL2A1	GATA3	IL6	MMP2	PRDM11	SIGIRR	UBE2I
BTNL8	COLQ	GBP4	ITGA4	MOV10L1	PRF1	SIGLEC12	VAMP5
C12orf65	CPVL	GIMAP1	ITK	MT1B	PRIMA1	SIRPB1	VNN1
C14orf177	CRCT1	GIMAP4	KCNE4	MTUS1	PRLR	SIRPB2	VSTM1
C17orf77	CRISPLD1	GIMAP5	KIF2B	MX2	PRR5	SLAMF8	VWA2
C17orf82	CST3	GIMAP7	KLRD1	NCALD	PRR5L	SLC12A8	WIPF1
C19orf45	CTNNA1	GIMAP8	KRT1	NDUFC2	PTPRB	SLC22A2	YES1
C20orf197	DEFA6	GMFG	KRT19	NEUROG1	RARRES3	SNORD105	ZFHX4
CALD1	DEFB135	GNA15	KRT78	NLRP12	RASSF1	SORBS1	ZNF117
CAPN14	DLG2	GNAS	KRT79	NLRP3	RD3	SPATA13	ZNF366
CCL5	DLX1	GPD2	KRT85	NMNAT2	RERE	SPN	ZNF718
CCR5	DLX4	GPM6A	KRTAP12-3	NOS1AP	RIMBP2	SST	
CD160	DOK2	GPR1	KRTAP19-8	NOX4	RNASE1	ST3GAL4	
CD177	ELAVL2	GPR132	KRTAP24-1	NPHP4	RNF150	STARD13	
CD226	EN1	GPR155	KRTAP6-3	NR2F2	RNF39	STBD1	
